# Supplementary material for: Radioprotective effect of the anti-diabetic drug metformin
Source: PLoS One. 2024 Jul 23;19(7):e0307598. doi: 10.1371/journal.pone.0307598 (PMC11265658; doi:10.1371/journal.pone.0307598)

S1B Fig

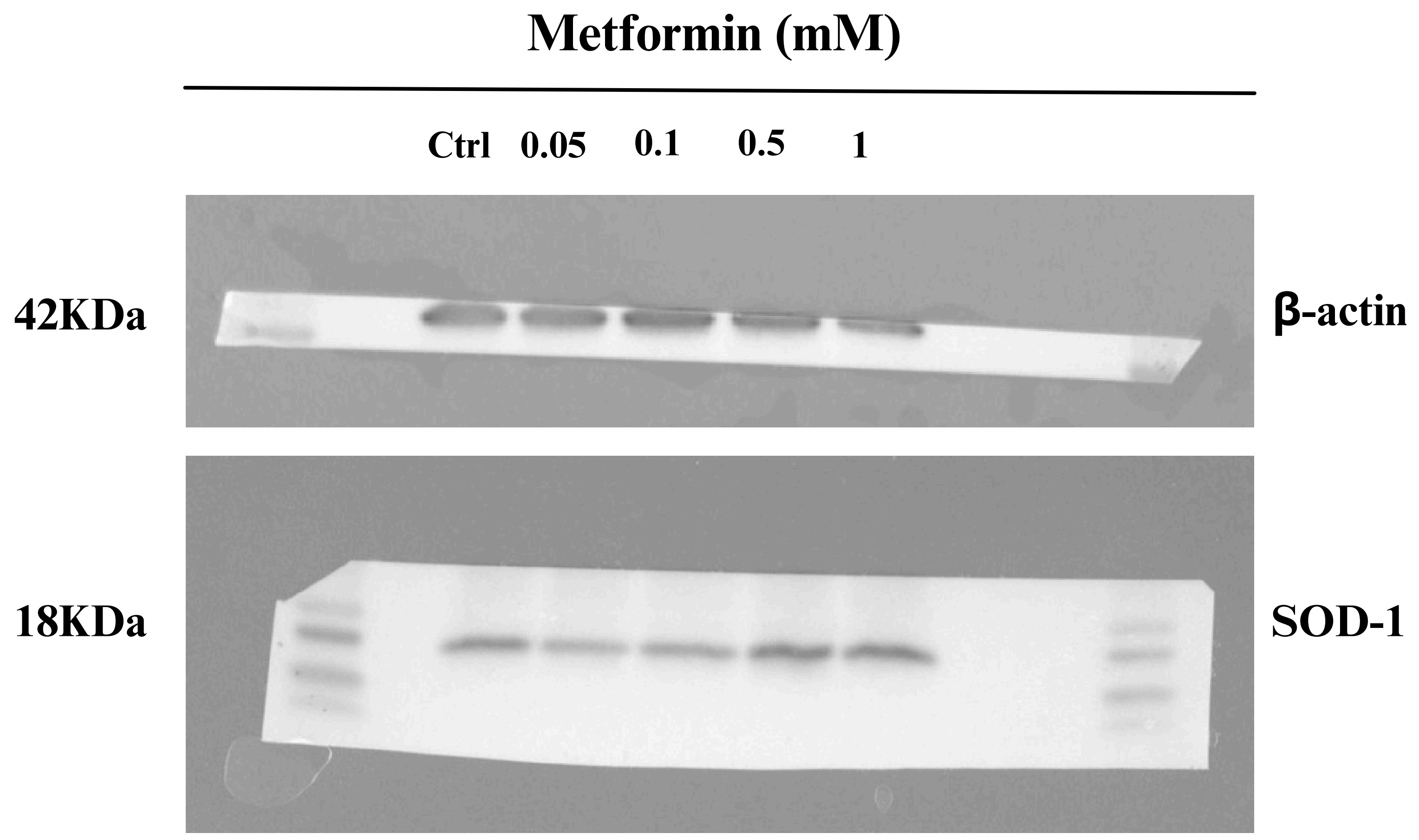

S1C Fig

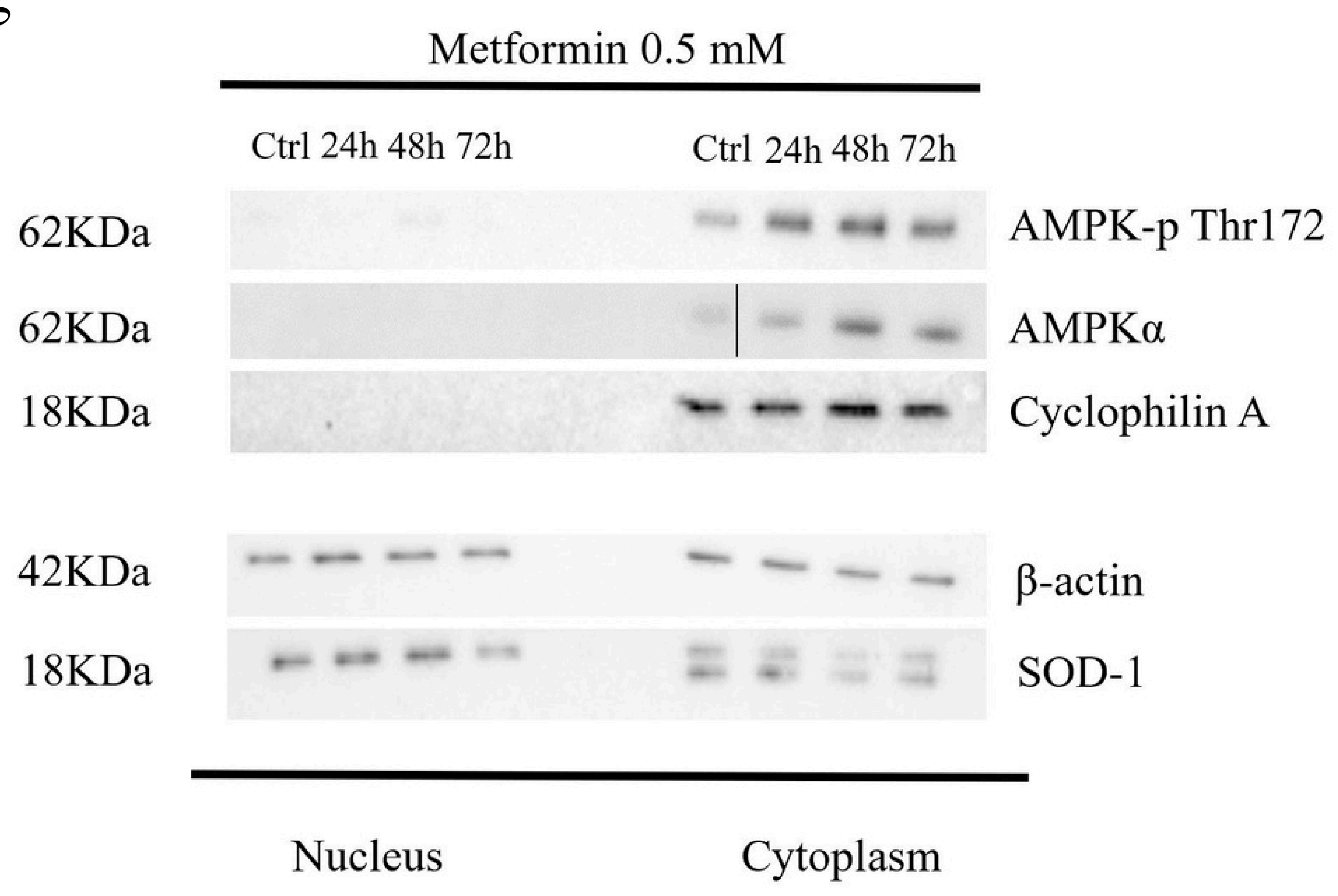

Original AMPKα from S1C Fig

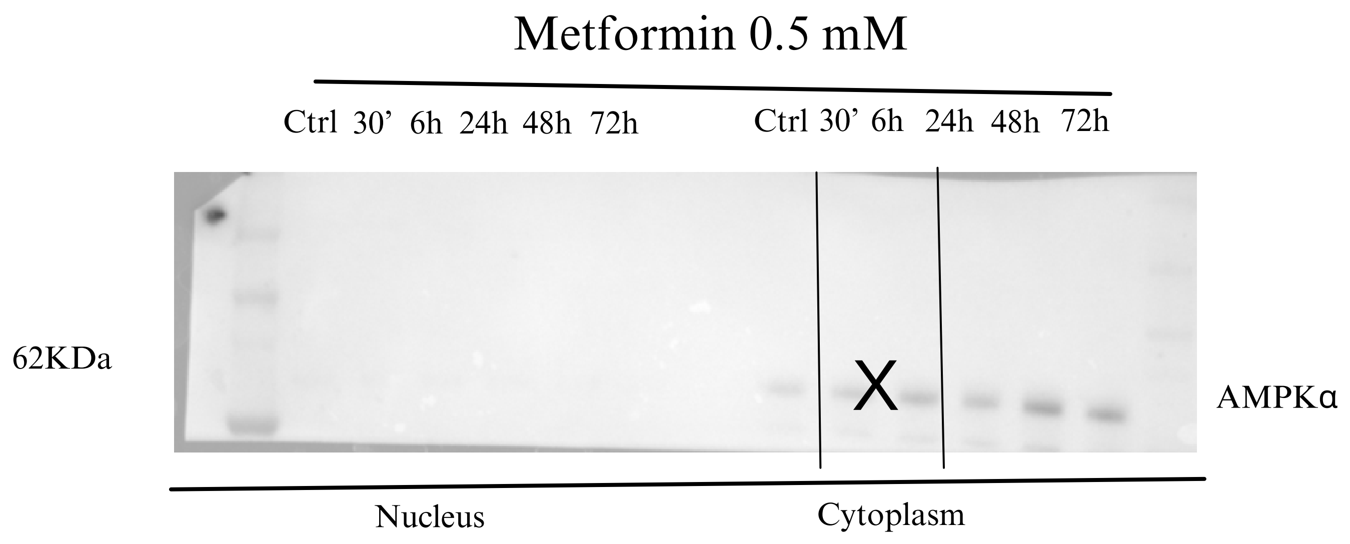

Original blots of S1C Fig

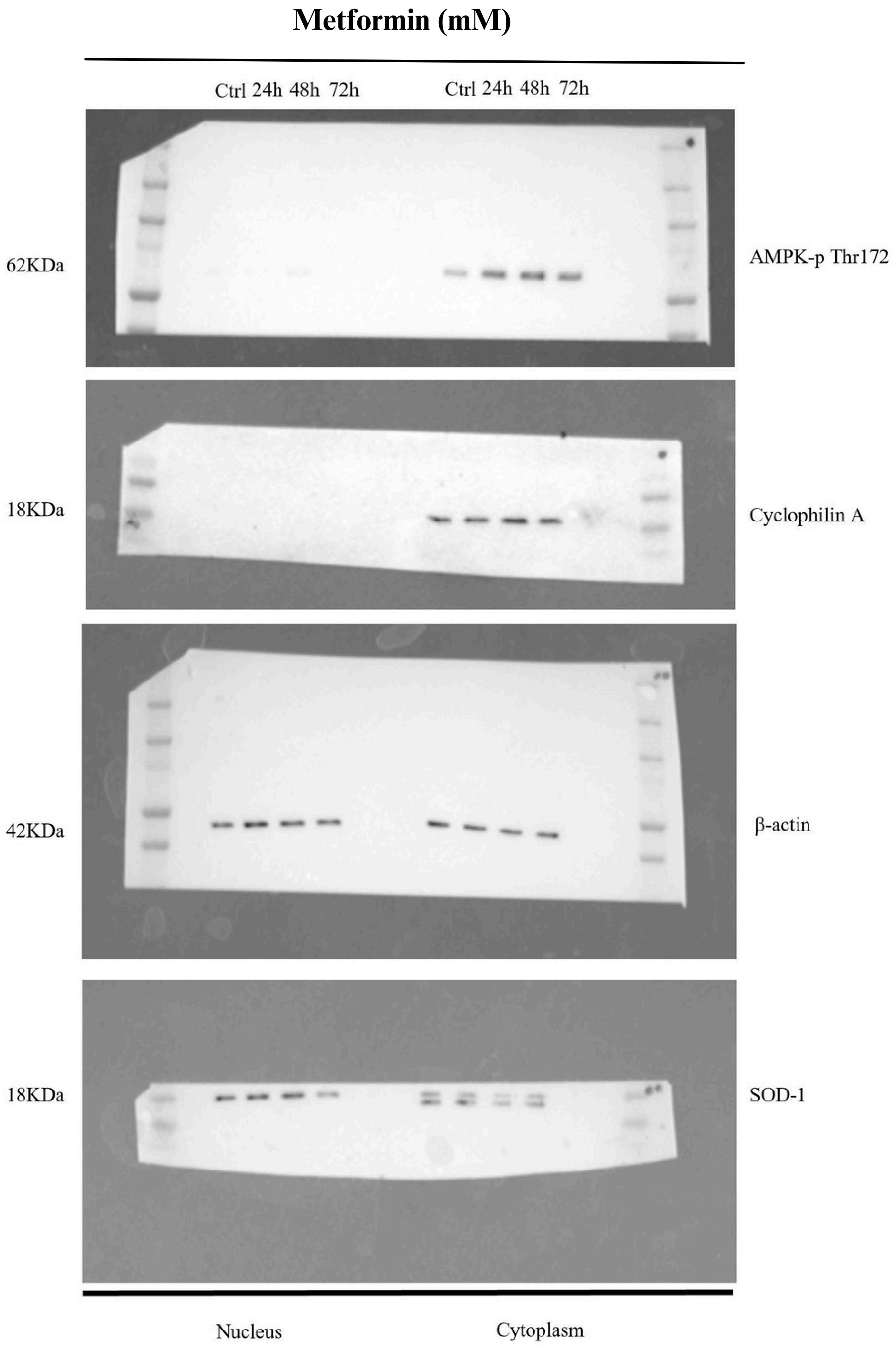

Fig 2A

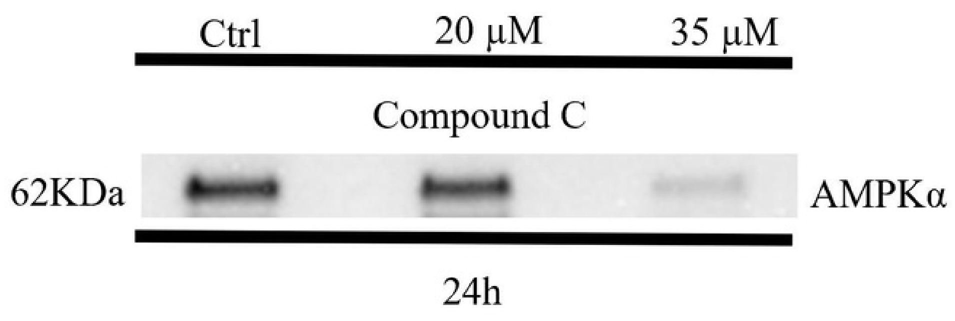

Original of Fig 2A

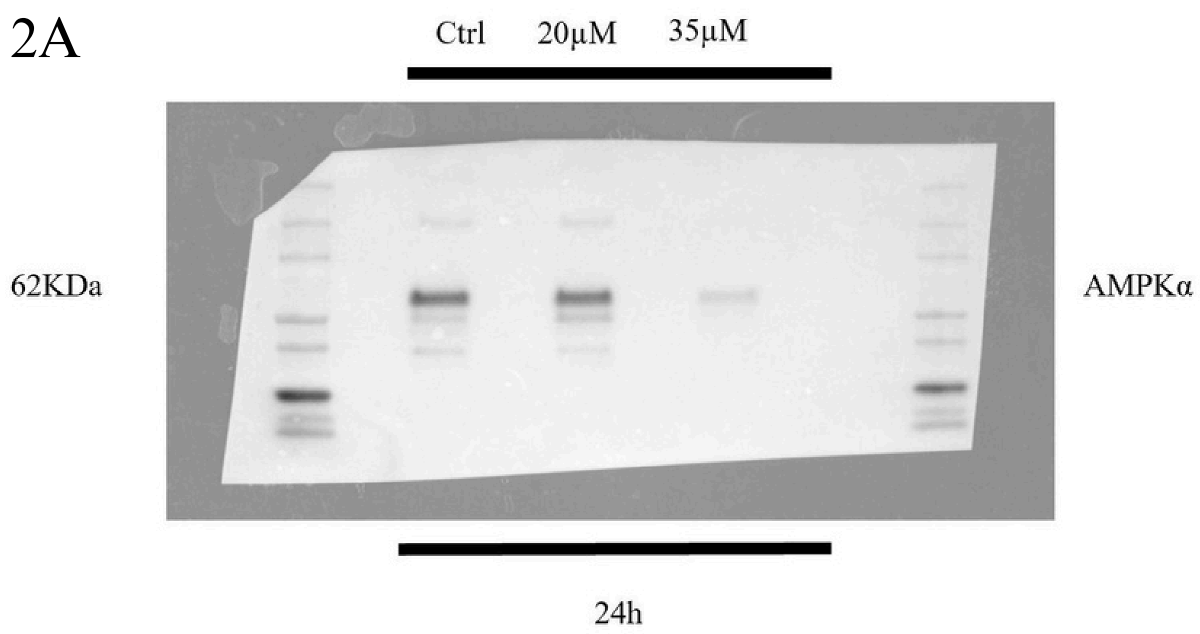

Fig 2B

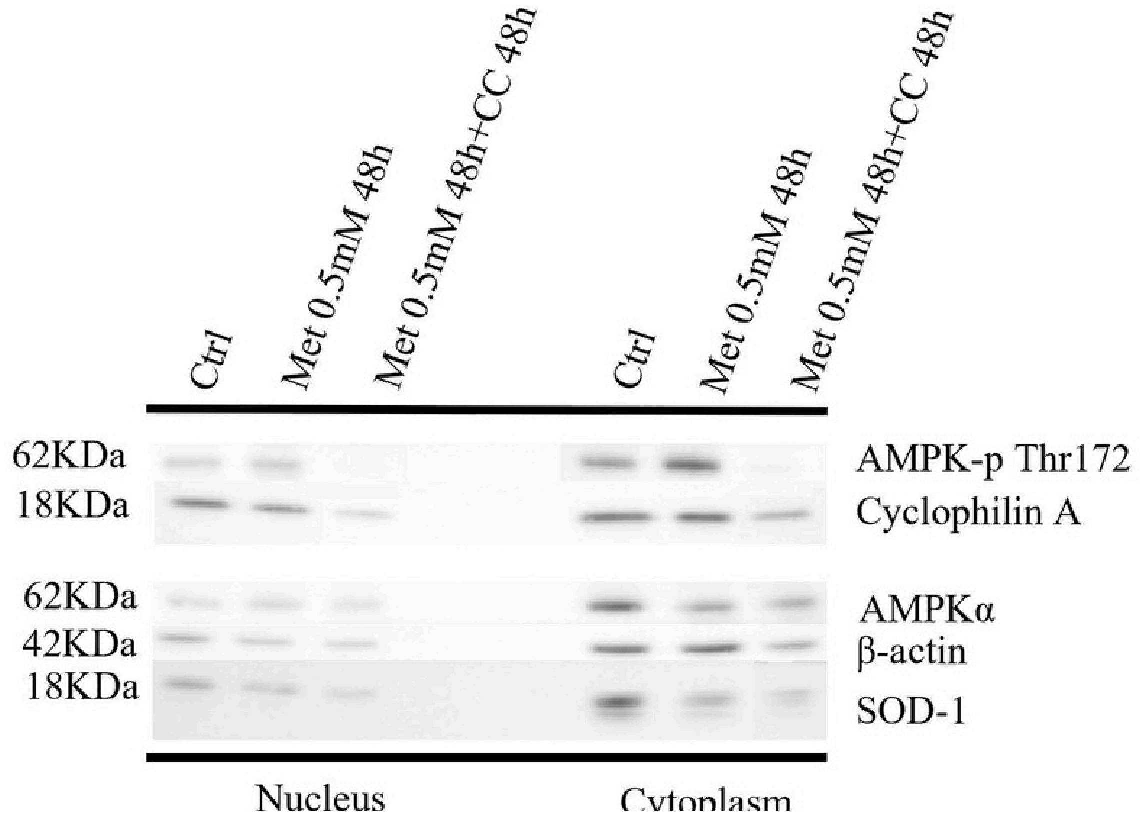

Original of Fig 2B

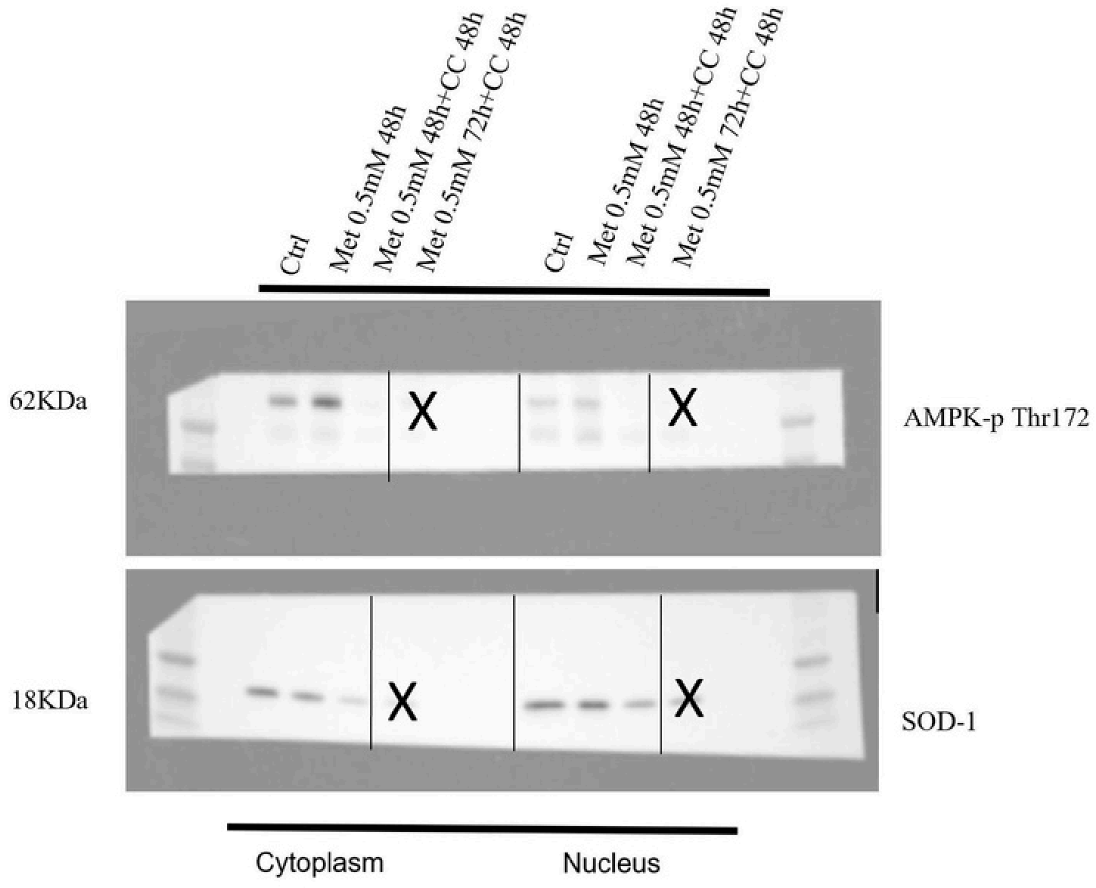

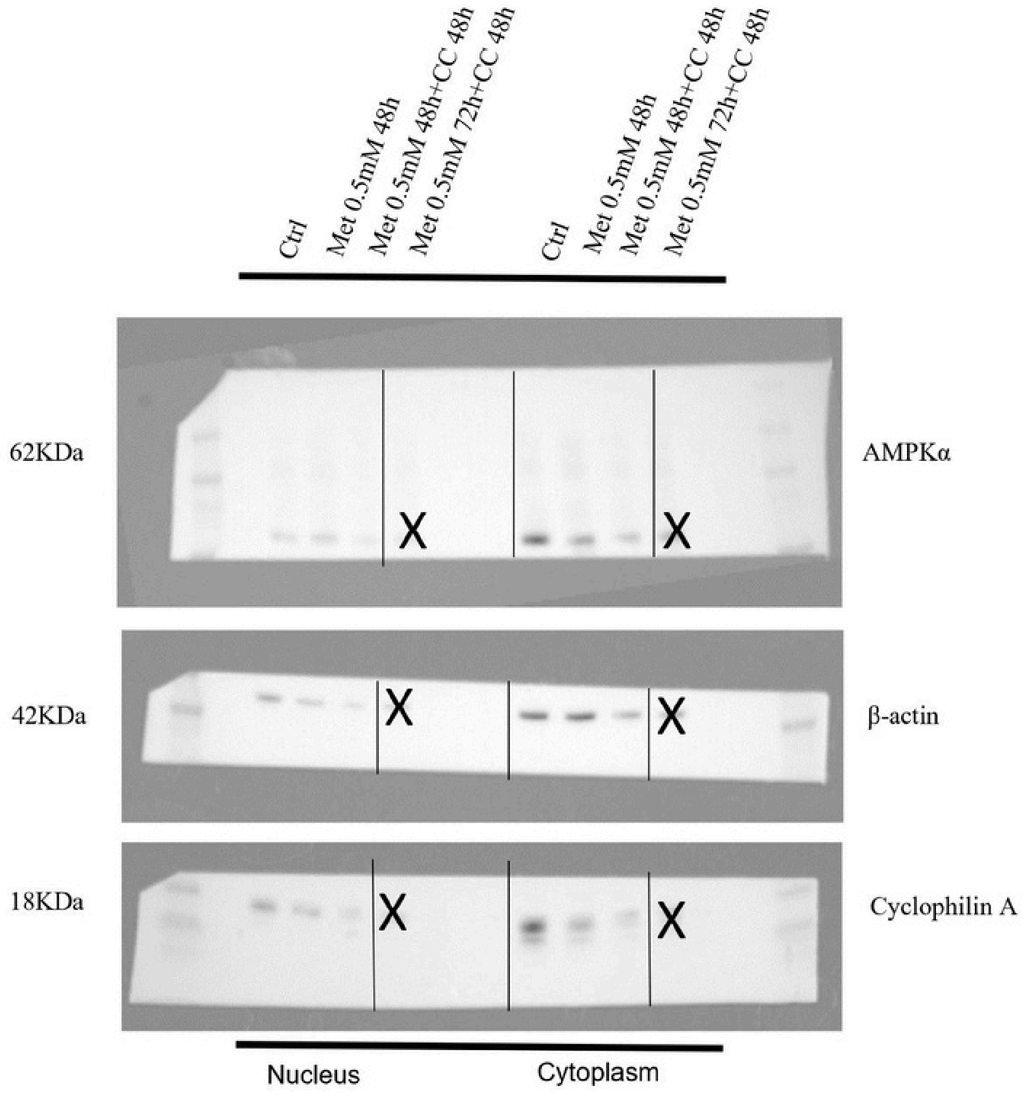

S4A

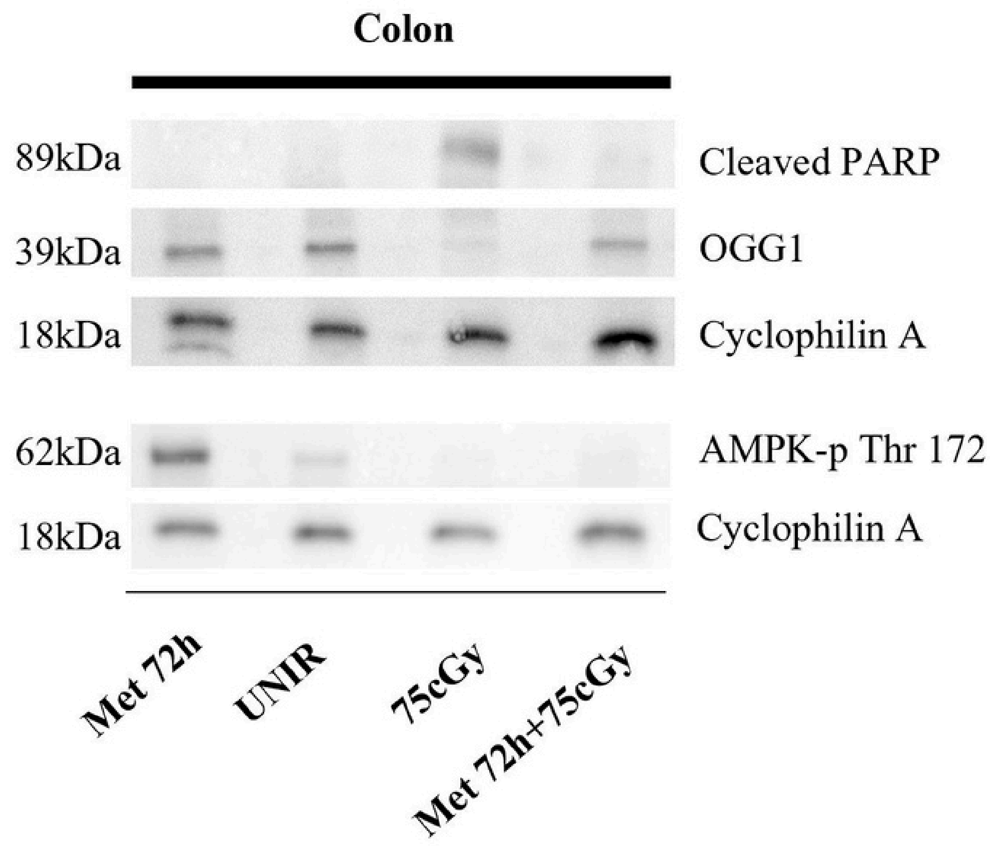

Original of S4A

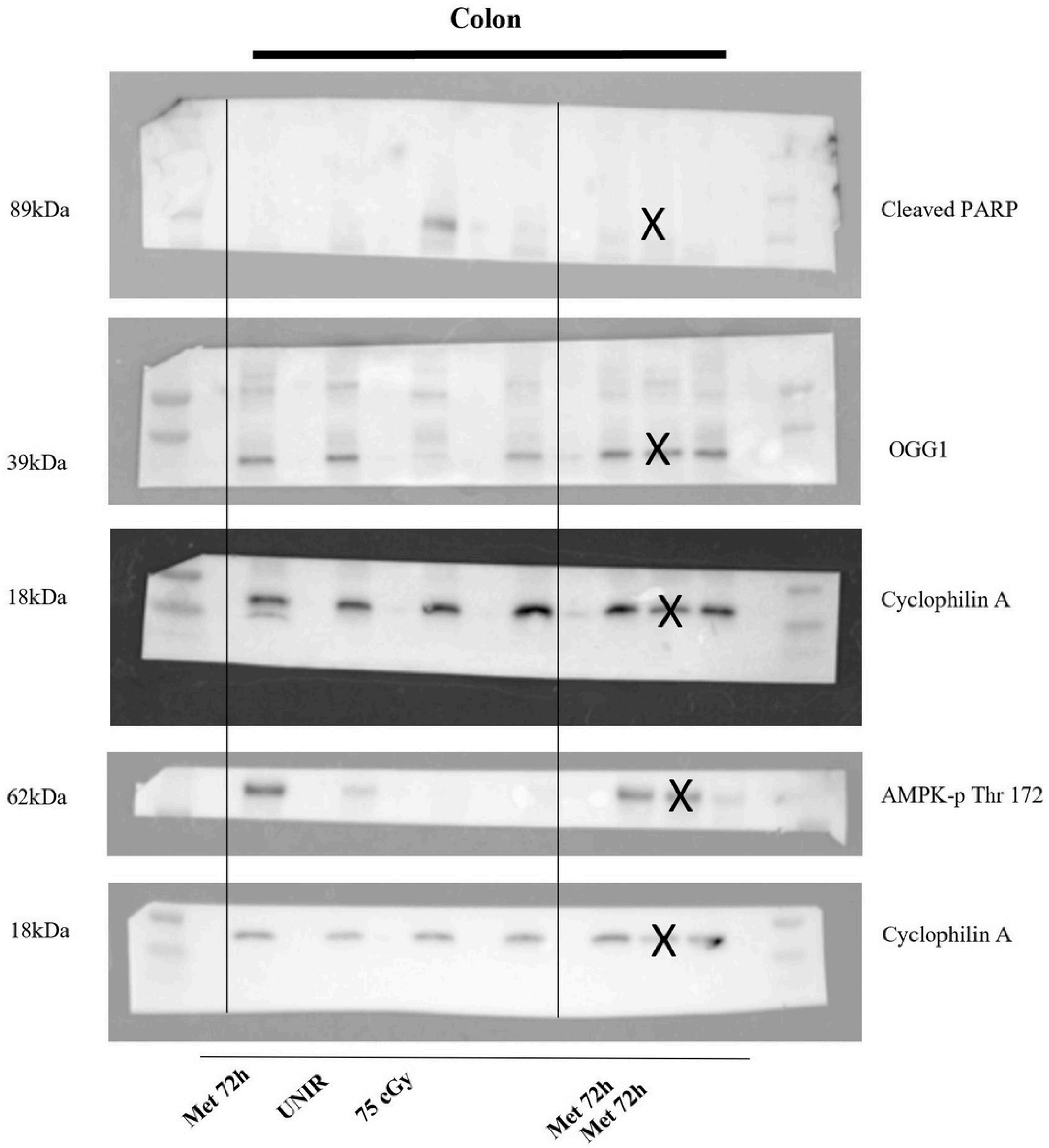

S4B

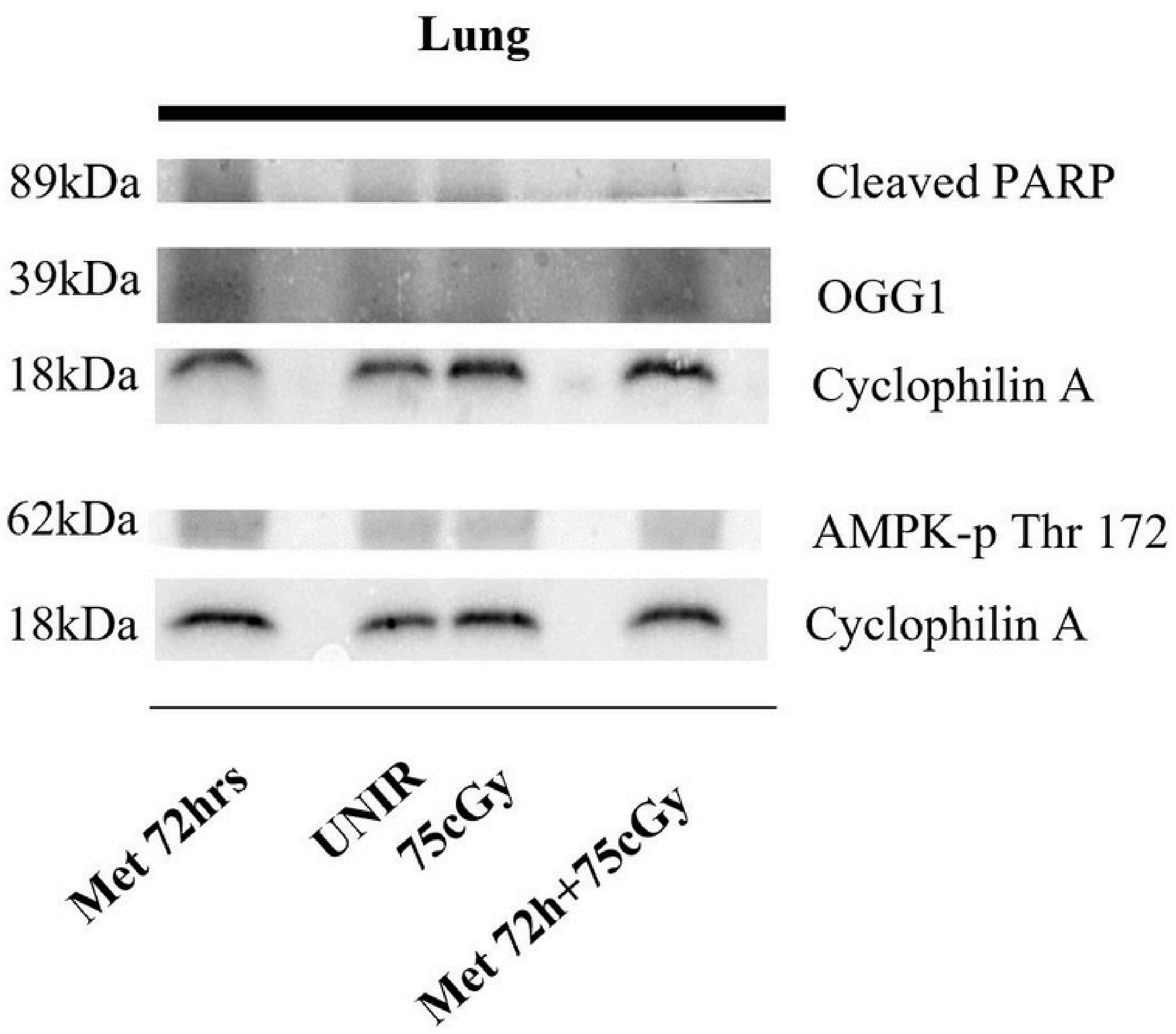

Original of S4B

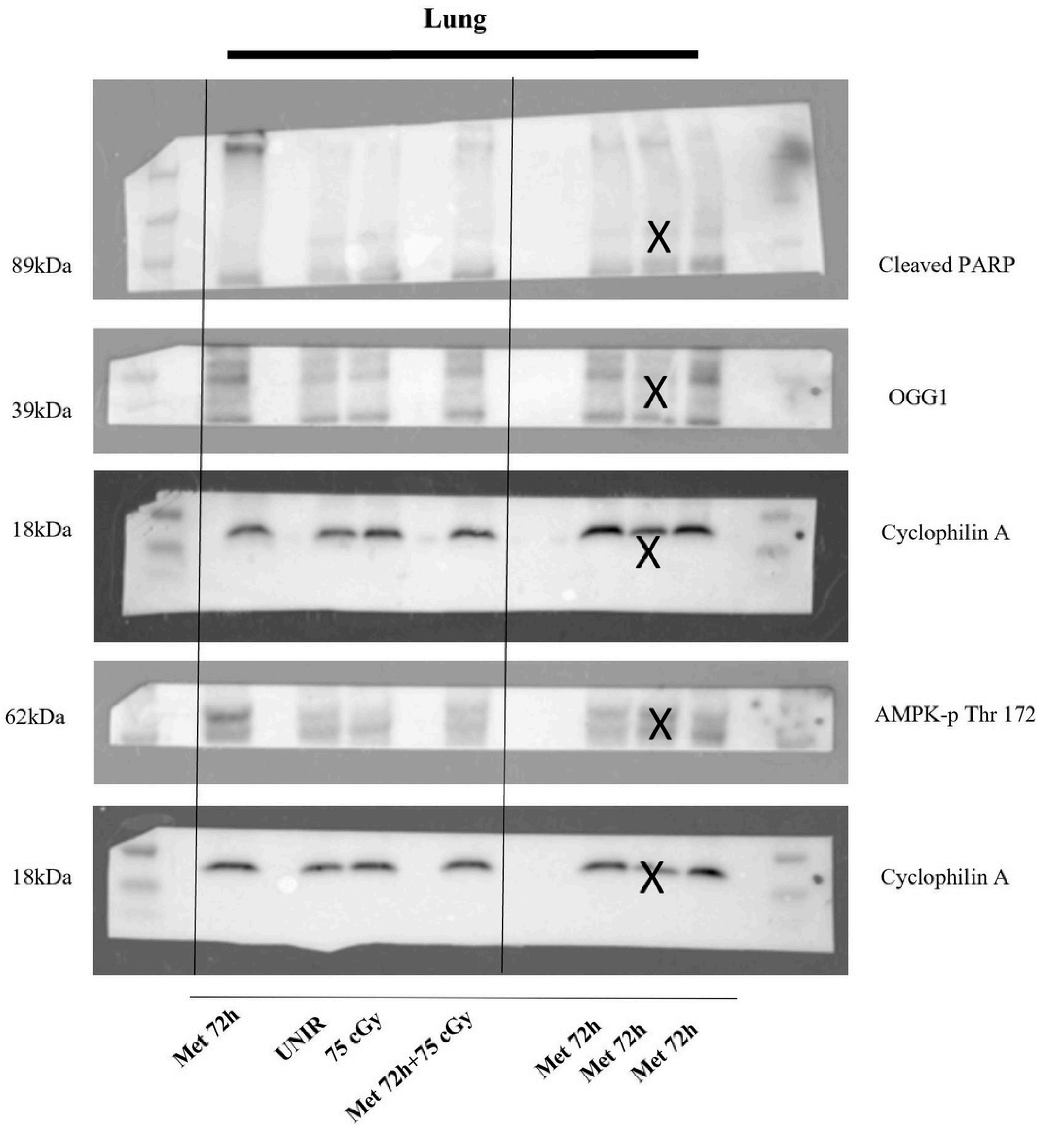

Supplement: S1 Raw images — (PDF) [file pone.0307598.s001.pdf]
