# Supplementary figures and images for: Radioprotective effect of the anti-diabetic drug metformin
Source: PLoS One. 2024 Jul 23;19(7):e0307598. doi: 10.1371/journal.pone.0307598 (PMC11265658; doi:10.1371/journal.pone.0307598)

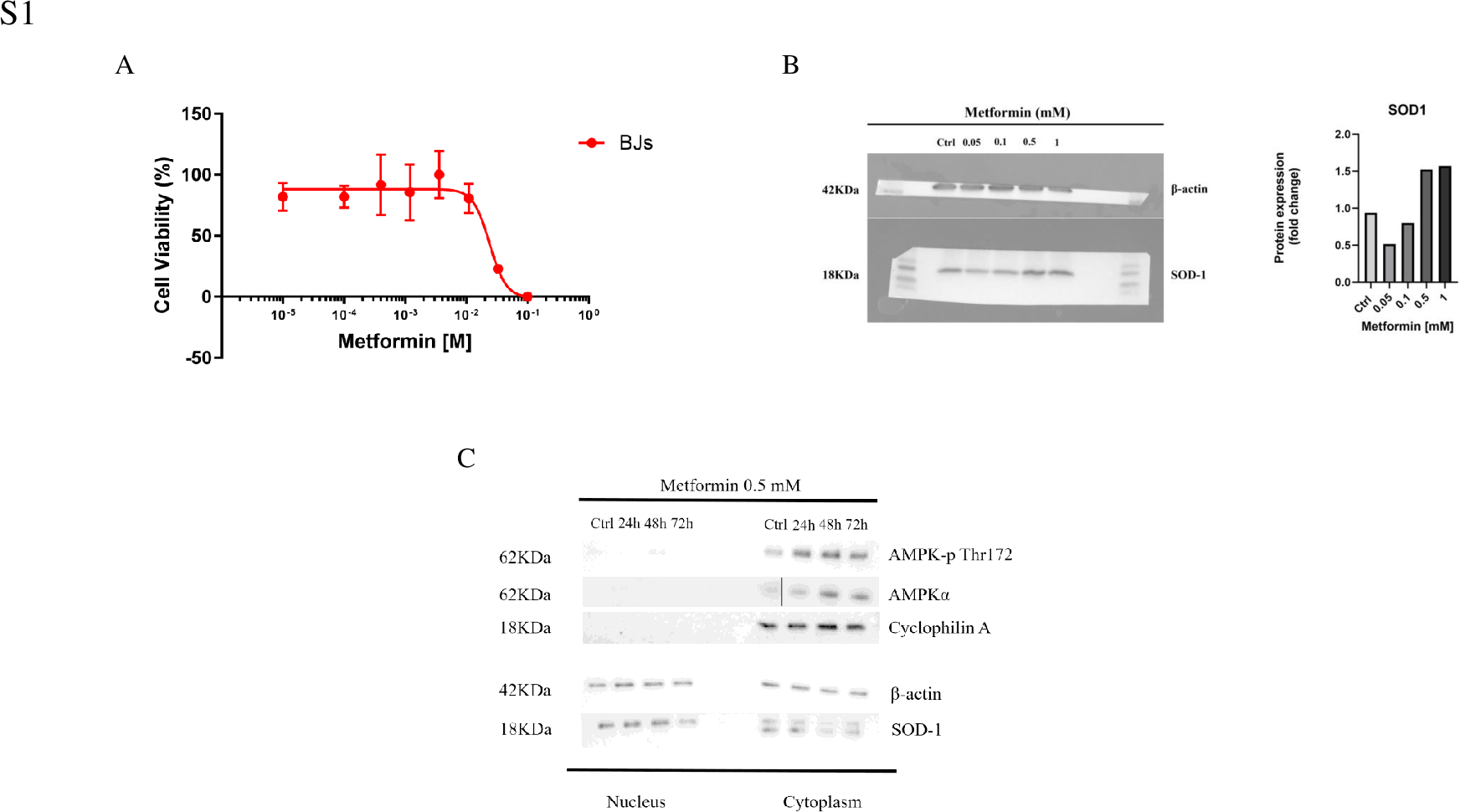

Supplement: S1 Fig — (A) Cell Titer Glo (CTG) analysis for the short-term effects of metformin on human BJs fibroblasts, IC50 = 25 mM. (B) Western blot showing maximun expression of SOD1 at 0.5 and 1 mM metformin, after one treatment only. (C) Western blot showing the increase expression of AMPK-p Thr 172 after one treatment only with metformin 0.5 mM, during the next 72 hours as well as an increase of SOD1 expression after 48 hours treatment in the nucleus. (TIF) [file pone.0307598.s002.tif]

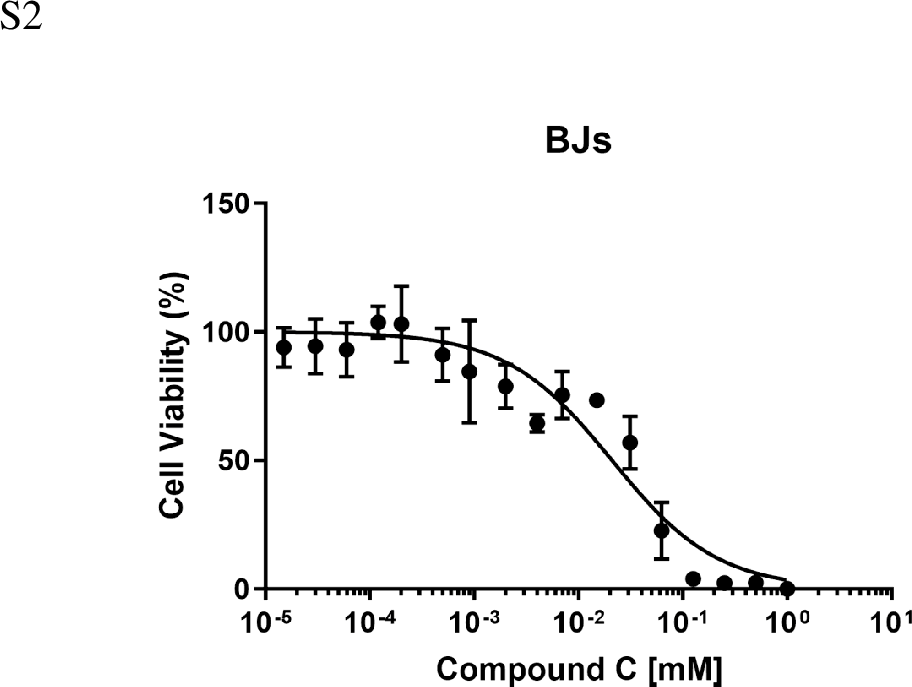

Supplement: S2 Fig — Cell viability curve in presence of different concentration of Compound C after 24 hours treatment in human BJ fibroblasts, IC50 = 35 μM. (TIF) [file pone.0307598.s003.tif]

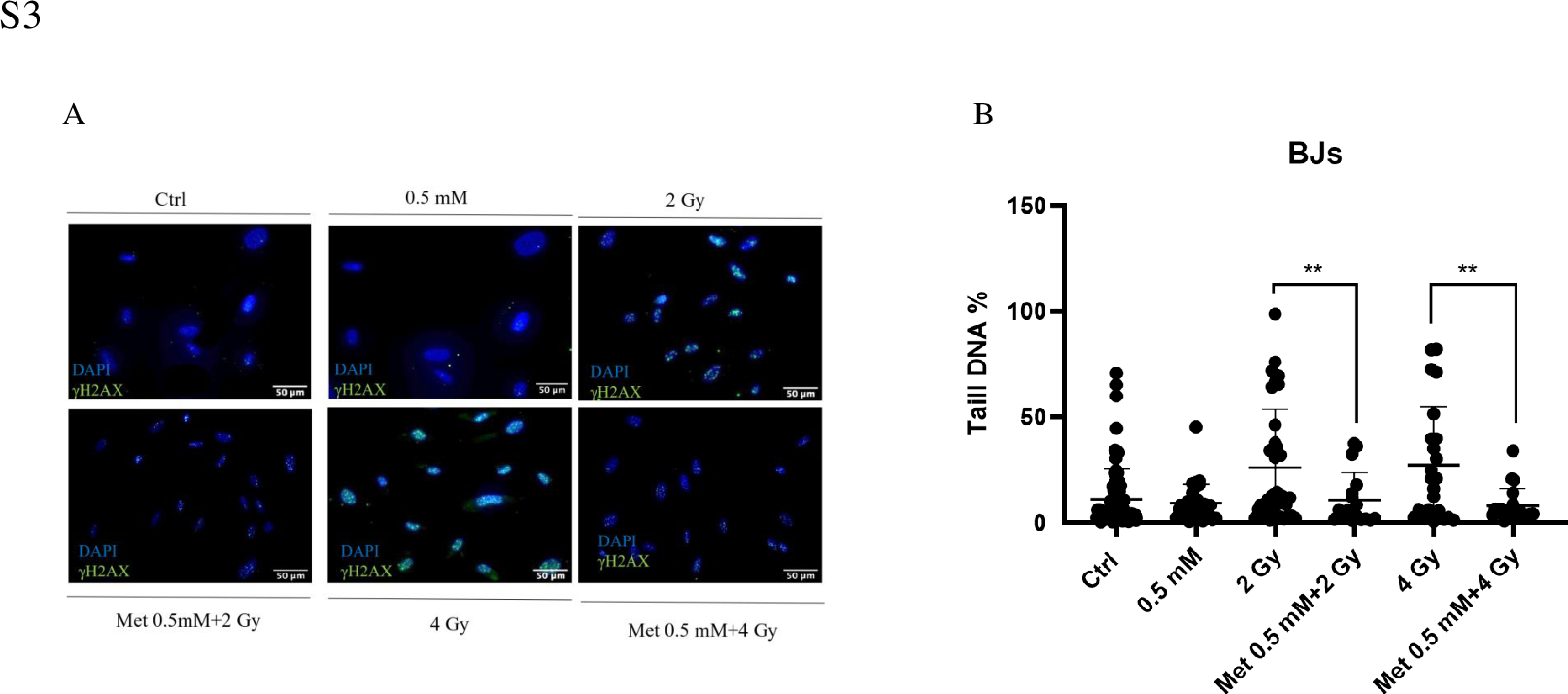

Supplement: S3 Fig — (A) Immunofluorescence of human BJs fibroblasts. Metformin 0.5 mM pre-treatment decreases the number of γH2AX foci in irradiated cells, compared to irradiated only controls. (B) Comet tail assay showing a decrease of the comet tail intensity in irradiated human BJ fibroblasts pre-treated with metformin 0.5 mM compared to the irradiated controls (student’s t-test, Welch correction, **p<0.01). (TIF) [file pone.0307598.s004.tif]

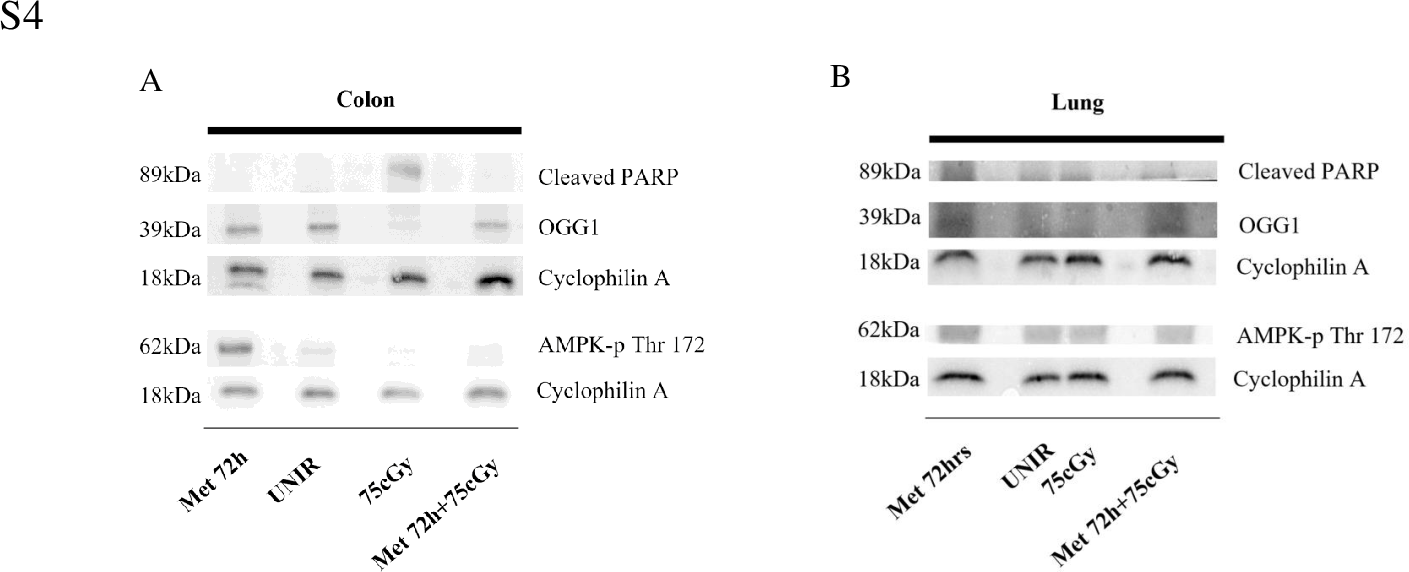

Supplement: S4 Fig — (A, B) Metformin retains the expression of OGG1 and decreases cleaved PARP expression in murine colon and lung tissues. Furthermore, metformin activates AMPK. (TIF) [file pone.0307598.s005.tif]
