# Supplementary material for: Radioprotective effect of the anti-diabetic drug metformin
Source: PLoS One. 2024 Jul 23;19(7):e0307598. doi: 10.1371/journal.pone.0307598 (PMC11265658; doi:10.1371/journal.pone.0307598)
Supplement: Attachment — Submitted filename: Response to Reviewers.docx [file pone.0307598.s006.docx]

**Review Comments to the Author**

We want to thank the editor and the reviewers for taking the time to read our paper and for the relevant questions.

Our responses to the initial review comments are detailed in the revised manuscript and summarized below.

**Reviewer #1**: In the current manuscript, the authors have repurposed metformin for its radioprotective effects, which are currently being used as a first-line treatment for managing type 2 diabetes. The authors utilized various in vitro and in vivo assays to explore its beneficial effects, including its potential use in space missions using galactic cosmic ray simulation (GCRsim). Additionally, the authors studied the possible mechanisms of radioprotection by metformin. In my opinion, the manuscript is well-written and organized.

However, I have several queries that the authors need to address in their revised version of the manuscript:

**1. In the determination of in vitro concentrations, the authors tested concentrations of 0.5, 1, 2.5, and 5 mM of metformin, ultimately deciding to use the lowest concentration of 0.5 mM. Why did they not explore even lower concentrations than 0.5 mM?**

Thank you very much for pointing out an important question. We did test lower concentrations of metformin and we focused on the lower concentrations of metformin able to induce the activation of SOD1. We added the data in the revised submission (S1 Fig B).

**2. For the in vivo study, the authors administered 200 mg/kg b.wt of metformin. How did the authors determine this concentration?**

Thank you for the question. We explained our choice in the material and methods section (p. 11, Drug preparation). Briefly, we evaluated the usual metformin administration in humans (between 1000 and 2000 mg per day). We used the body surface area (BSA) normalization method [53] according to the FDA guidelines [54] and we found a minimal dosage of 250 mg/kg. However, since our aim was to study the radioprotective effects of metformin in healthy wild type mice (and potentially in very healthy human beings such as astronauts) we decreased the concentration to 200 mg/kg. We explained this better in the revised manuscript

**3. What is the LD50/30 dose for 129/sv mice? I ask this because researchers generally use sub-lethal doses for mechanistic studies and slightly higher doses than the LD50/30 for survival studies. In this study, the authors used 7.5 Gy for mechanistic studies and 10 Gy for survival studies.**

According to previous studies in our lab and others, wild type mice are sensitive to 7.5 Gy of total body x-rays. However, when we did a pilot test, our mice were not lethally affected by 7.5 Gy, but most wild type mice died after two weeks when irradiated once with 10 Gy x-rays. We used the sublethal dose of 7.5 Gy to better investigate the DNA repair kinetics and the lethal dose of 10 Gy to evaluate the overall survival in the presence or absence of metformin.

**Reviewer #2**: The manuscript presents a comprehensive study investigating the potential radioprotective effects of metformin and its underlying mechanisms. Here's a review based on the provided criteria:

Technical Soundness and Data Support for Conclusions: The manuscript appears technically sound, with a detailed exploration of the effects of metformin on various cellular processes, particularly in response to ionizing radiation. The authors provide data on AMPK activation, changes in antioxidant enzyme expression, DNA repair kinetics, and survival outcomes in both in vitro and in vivo models. The findings suggest that metformin pre-administration confers radioprotection by enhancing DNA repair mechanisms and mitigating cellular damage induced by ionizing radiation. However, while the data presented support the conclusions drawn, **there are some areas where further clarification or additional experiments may strengthen the claims. For instance, it would be beneficial to include more detailed analyses of the molecular pathways involved in metformin-mediated radioprotection, such as downstream targets of AMPK activation and the interplay between different cellular processes.**

Thank you for appreciating our work and paper. We agree with the reviewer about including a deeper investigation into the mechanisms to elucidate the role of AMPK effects downstream. We did investigate the main pathways involving the DNA damage response and AMPK. Below is what we discussed in the original draft that addresses this comment:

*“…we found metformin 0.5 mM increases the phosphorylation of ataxia telangiectasia mutated (ATM) at serine 1981 and p53 expression after 48 hours. ATM is a kinase that acts upstream of p53 and controls a DDR pathway critical to resolving DSBs [45]. However, we did not find any increase in γH2AX foci in vitro or in 53BP1 foci in vivo, but this might be related to the time-points we chose to analyze. Vazquez-Martin et al. [46] reported ATM phosphorylation at serine 1981 and formation of γH2AX foci after metformin exposure in epidermoid carcinoma cells, but not of 53BP1. This is in line with what was reported by Bakkenist & Kastan [47], showing ATM activation is not dependent on direct binding to DNA strand breaks, but may result from changes in the structure of chromatin. Interestingly, the increased expression of p53 might have effects on the cell cycle, inducing a transient arrest in G1 phase, and thus providing time for DNA repair from the effects of IRs*.”

We did not obtain significant data about CHK1, CHK2, BRCA1, ATR expression and these results are not included in the revised manuscript.
